# Communicating with parents about vaccination: a framework for health professionals

**DOI:** 10.1186/1471-2431-12-154

**Published:** 2012-09-21

**Authors:** Julie Leask, Paul Kinnersley, Cath Jackson, Francine Cheater, Helen Bedford, Greg Rowles

**Affiliations:** 1 School of Public Health, and Discipline of Paediatrics and Child Health University of Sydney and National Centre for Immunisation Research and Surveillance, The Children’s Hospital at Westmead, Sydney, Australia; 2 Institute of Primary Care and Public Health, Cardiff University, 3rd floor, Neuadd Meirionydd, Heath Park, Cardiff, CF14 4XN, UK; 3 York Trials Unit, Department of Health Sciences, University of York, York, YO10 5DD, UK; 4 Institute for Applied Health Research, Associate Dean (Research) School of Health and Life Sciences, Buchanan House, Glasgow Caledonian University, Glasgow, G4 OBA, UK; 5 Centre for Paediatric Epidemiology and Biostatistics, UCL Institute of Child Health, 30 Guilford Street, London, WC1N 1EH, UK; 6 Greg Rowles, General practitioner, 9 Station St, Riddells Creek, VIC, 3431, Australia

## Abstract

**Background:**

A critical factor shaping parental attitudes to vaccination is the parent’s interactions with health professionals. An effective interaction can address the concerns of vaccine supportive parents and motivate a hesitant parent towards vaccine acceptance. Poor communication can contribute to rejection of vaccinations or dissatisfaction with care. We sought to provide a framework for health professionals when communicating with parents about vaccination.

**Methods:**

Literature review to identify a spectrum of parent attitudes or ‘positions’ on childhood vaccination with estimates of the proportion of each group based on population studies. Development of a framework related to each parental position with determination of key indicators, goals and strategies based on communication science, motivational interviewing and valid consent principles.

**Results:**

Five distinct parental groups were identified: the ‘unquestioning acceptor’ (30–40%), the ‘cautious acceptor’ (25–35%); the ‘hesitant’ (20–30%); the ‘late or selective vaccinator’ (2–27%); and the ‘refuser’ of all vaccines (<2%). The goals of the encounter with each group will vary, depending on the parents’ readiness to vaccinate. In all encounters, health professionals should build rapport, accept questions and concerns, and facilitate valid consent. For the hesitant, late or selective vaccinators, or refusers, strategies should include use of a guiding style and eliciting the parent’s own motivations to vaccinate while, avoiding excessive persuasion and adversarial debates. It may be necessary to book another appointment or offer attendance at a specialised adverse events clinic. Good information resources should also be used.

**Conclusions:**

Health professionals have a central role in maintaining public trust in vaccination, including addressing parents’ concerns. These recommendations are tailored to specific parental positions on vaccination and provide a structured approach to assist professionals. They advocate respectful interactions that aim to guide parents towards quality decisions.

## Background

The benefits of childhood vaccination are well established [1]. Vaccine uptake rates in most industrialised countries are generally high. However, two broad parental factors are associated with under-vaccination. The first relates to socioeconomic disadvantage where, despite some motivation to have their children vaccinated, parents or carers (hereafter referred to as ‘parents’) lack access to adequate resources and support to overcome logistical barriers such as a lack of transport or childcare [2,3]. The second factor, and the focus of this paper, relates to parents’ concerns about the safety or necessity of vaccines [4,5].

A critical factor shaping parental attitudes to vaccination is the parents’ interactions with health professionals. An effective interaction can address the concerns of vaccine supportive parents and motivate a hesitant parent towards vaccine acceptance [5,6]. Conversely, poor communication can contribute to rejection of vaccinations or dissatisfaction with care [7-9]. Such poor communication often results from a belief by the health professional that vaccine refusal arises from ignorance which can simply be addressed by persuading or providing more information. Such an approach is counter-productive because it fails to account for the complexity of reasons underpinning vaccine refusal and may even result in a backfire effect [10]. Parental vaccination decisions are based on an array of factors and parents integrate information according to their experiential and social contexts [11,12]. A parent’s trust in the source of information may be more important than what is in the information [13,14].

Health professionals have a central role in maintaining public trust in vaccination; this includes addressing parents’ vaccine concerns [15]. These concerns will likely increase as vaccination schedules inevitably become more complex, and parents have increased access to varied information through the internet and social media [16]. In recognition of the need to support health professionals in this challenging communication task conducted in usually short consultations, recommendations have been proposed [17-21]. Previously, most of these have focused on *what* is said, that is, the information that should be given to parents. Few have addressed *how* health professionals should engage with parents [17-21]. Since it is clear that parents want an improved dialogue about vaccinations [22-24], it is essential to focus on communication processes that build rapport and trust between the health professional and the parent [25-27].

We propose here a framework to guide health professionals in communicating with parents about vaccination. By focusing on both what is said and how it is said, we attempt to provide an integrated, generic approach going beyond simply the one-way provision of information.

The framework is informed by evidence from decision making and communication research and is applicable for use by all health professionals in their vaccination discussions, particularly where there might be parental reluctance to vaccinate. It focuses on recommended childhood vaccines but is also applicable to discussions with other groups recommended for vaccination. We propose categories or ‘positions’ that reflect different parental attitudes and behaviours regarding vaccination and suggest specific communication strategies tailored to each position. The overarching goal of the encounter is to promote quality decisions and, ultimately, vaccination.

### Development of the framework

The framework was developed to:

a) be acceptable to health professionals

b) increase health professional and parent satisfaction with discussions about vaccination

c) increase a health professional’s self-efficacy (sense of confidence and competence) in relation to communicating about vaccination

d) increase the likelihood of the parent making a decision based on evidence (by increasing access to quality information)

e) encourage uptake of recommended vaccines.

There were four stages in developing the framework: a literature review, classifying parental positions on vaccination, matching strategies to these positions and assessing their face validity with heath professionals.

#### Stage 1: Literature review

This aimed to (1) identify existing research that had classified parents’ positions based on their attitudes and behaviours regarding childhood vaccination and (2) identify articles that contained strategies to communicate with parents about vaccination. We searched MEDLINE (1996–), PsycINFO (1967–), CINAHL (1982–), and EMBASE (1980–) in September 2010. The following combination of keywords and associated MESH headings (identified for each database) was used: child$ or infant$ or newborn$ or baby or babies AND vaccin$ or immunis$ or immuniz AND decision$ or choice behavio$ or choic$ or communicat$ or consult$.

The searches identified 3168 total hits (including duplicates) which was reduced to 112 after screening titles and removing duplicates. Of these, we identified three studies that proposed a spectrum spanning the parental positions on vaccination. These are described in Table 1[28-30]. We found nine papers advising health professionals on communicating with patients about vaccination but none that tailored communication to empirically derived parental positions [17-21,31-34]. 

**Table 1:** Summary of studies identifying parental positions on vaccination

**Study**	**Setting/sample**	**Method**	**Results**
Gust et al., 2005[28]	US population-based sample of 584 parents with at least one child aged 6 years and under (The ConsumerStyles and HealthStyles surveys 2002)	Telephone administered questionnaire.	3.9% reported that child had not had all recommended immunisations.
		44 questions about beliefs and attitudes towards vaccination, influence of family and friends on vaccination decisions and dependence on doctor’s advice. K-means cluster ANOVA analysis to group like responses.	Five attitudinal categories:
			‘immunisation advocate’ (33%); ‘go along to get along’ (26%); ‘health advocate’ (25%); ‘fence sitter’ (13%); and ‘worried’ (2.6%).
Downs et al., 2007[30]	30 US parents of children aged 18–23 months, recruited from three cities with diverse socio-demographic profiles and vaccination attitudes	Mixed methods ‘mental models’ interviews conducted by telephone. Open and closed ended questions were designed to identify predominating cognitive pathways in decision making about vaccination.	Two main decision making types although views were overlapping:
			‘health oriented’ (n = 16) trusted anecdotal communication more than statistical arguments;
			‘risk oriented’ (n = 14) trusted communication with statistical arguments more than anecdotal information.
Benin et al., 2006[29]	33 US mothers recruited post partum in one hospital or in the care of participating midwifery practices in one US state	All mothers were interviewed face to face in immediate postpartum period and 19 mothers were interviewed by telephone when baby was 3–6 months old to determine attitudes towards vaccinating; risks and benefits of vaccination; and requirements for, and sources of, information. Respondents categorised into groups, based on behaviours and attitudes.	Two main categories – Vaccinators (n = 25) with sub-categories: ‘acceptors’ (n = 20) and ‘vaccine hesitant’ (n = 5).
			Non-vaccinators (n = 8) with subcategories: ‘late (or partial) vaccinators’ (n = 3) and ‘rejectors’ (n = 5) who refused all vaccines.

#### Stage 2: Identifying parental positions on vaccination

We reviewed the classifications presented within the three identified studies, [28-30] summarised parental positions from each study, and discussed their relevance to vaccine communication. A discrete number of parental positions relating to vaccination attitudes and behaviour were proposed, discussed and revised based on the categories’ applicability to clinical interactions and international relevance. The final set of five parental positions are described in Table 2. A range for the approximate proportion of each group is given based on population surveys or registers from the USA, European Union, New Zealand, and Australia [4,28,35-44]. 

**Table 2:** Parental positions on vaccination according to attitudes and behaviours

**Unquestioning acceptor 30–40%**	These parents vaccinate, or want to, vaccinate their children and have no specific questions about the safety and necessity of vaccines. In Gust’s study, they corresponded with the ‘immunisation advocates’ or ‘go along to get along’ groups who see the importance of childhood vaccination and are confident in its safety [28]. They report a good relationship with their healthcare provider and agree that medical professionals have their child’s best interests at heart. This group tend to have less detailed knowledge about vaccination [29,30].
**Cautious acceptor 25–35%**	These parents vaccinate their children despite minor concerns. They may exhibit a ‘hope and pray’ mentality recognising that vaccines carry rare but serious side effects and hoping that their child is not affected [45]. Both this category and ‘unquestioning acceptors’ were drawn from Benin’s category of ‘vaccination acceptors’ [29].
**The hesitant 20–30%**	These parents vaccinate their child but have significant concerns [29]. In Gust’s study, they most closely correspond to the ‘fence-sitter’ who only slightly agrees about the benefits and safety of vaccination and is neutral about their relationship and trust with their healthcare provider [28]. Hesitants are also more focused towards vaccine risk, and are aware of issues surrounding the MMR vaccine and of other parents not vaccinating their children [30]. Trust in their doctor or nurse is key for this group who are keen to have discussions in which their questions are answered satisfactorily and completely by knowledgeable health professionals with relevant information [29].
**Late or selective vaccinator 2–27%**	Concerns about vaccination result in this group choosing to delay or select only some recommended vaccines [38]. This group most closely correspond with Gust’s ‘worried’ category with significant doubts about the safety and some doubt about the necessity of vaccines [28]. They have concerns about the number of vaccines children have [39]. They experience conflicting feelings about how to get their questions answered and who to trust, [39] and are similar to the vaccine hesitant in actively seeking information [29,46]. Probably because they actively seek information, in Benin’s study they had the highest levels of knowledge about vaccination [29] and in Downs' study prefer red statistical arguments to anecdotal information [30]. With a specific vaccine scare hesitant parents may ‘select-out’ the vaccine and move to this category, as was the case with MMR vaccine in the UK [40].
**Refuser <2%**	Parents in this group refuse all vaccines for their child. This results from either their existing philosophical position on vaccination, negative experiences with the medical system, or religious beliefs [9]. Contact with the medical establishment and doctors often results in feelings of alienation and disenchantment and they tend to prefer the advice of alternative health professionals [29,41]. Respondents in Benin’s study indicated a desire for a doctor with whom they could enjoy a trusting relationship and who would accept their decisions about vaccination. Benin’s sample of 33 mothers had less accurate knowledge about vaccination than all other groups except ‘acceptors’ [29]. These parents tend to cluster in communities who share certain religious, philosophical or alternative beliefs [47].

#### Stage 3: Matching strategies to parental positions

For each parental position, we proposed an overall communication approach then more specific guidance tailored to each parental position (1 3 and 4). Given that no tailored guidance was identified in the literature review, the specific strategies were informed by the literature on health communication; [48,49] our professional and educational experience; valid consent principles, [50] and those of motivational interviewing which uses a guiding style to promote healthy behaviours [51].

#### Stage 4: Seeking feedback from health professionals

To assess general acceptability of the framework and recommendations, accredited nurse immunisation providers were presented with them in three annual update sessions, and 104 completed a short questionnaire about usefulness (scored from 1 ‘not at all useful’ to 10 ‘extremely useful’), realism (not at all/somewhat/very), strengths (open question), and areas for improvement (open question). Feedback was positive with a median score for usefulness of 8.8 (range 3–10); 74% rated the framework as ‘very realistic’, 26% as ‘somewhat realistic’, while no-one rated it as ‘not at all realistic’. General practice immunisation coordinators, immunisation experts, and a consumer representative (n = 20) all provided verbal feedback on the draft framework. All feedback was used to inform revisions of the recommendations.

### The framework

#### How discussions are addressed

While the majority of parents accept vaccination (Table 2), attendance at the consultation should not be presumed to indicate consent. Ideally parents will receive credible information prior to their child’s appointment. Health professionals have a responsibility to ensure that parental consent for vaccination is valid. This requires more than simply giving information and is built upon a relationship and interaction [50,52].

Building trust is paramount in any healthcare interaction. As noted by Benin et al, the trusted health professional is one who: has spent time with the child and parent; listened to, accepted and addressed their concerns; possesses the necessary scientific information; and uses a whole-person approach that is not patronising but treats parents and their children as individuals [29]. Table 3 lists unhelpful and helpful approaches to communication with all parents. 

**Table 3:** Unhelpful and helpful strategies for addressing parental concerns about vaccination

**Unhelpful**	**Helpful [**[51]**]**
**Directing style – “this is what you should do”**	**Guiding style – “may I help you?”**
Righting reflex – using information and persuasion to achieve change	Care with body language
Missing cues	Eliciting concerns
Using jargon	Asking permission to discuss
Discrediting information source	Acknowledging/listening/empathising
Overstating vaccine safety	Determining readiness to change
Confrontation	Informing about benefits *and* risks
	Giving or signposting appropriate resources

Health professionals’ body language ideally indicates that the discussions are important and distractions, such as using computers while talking, are best avoided. They need to speedily establish rapport and clarify parental concerns, avoiding the temptation to minimise or dismiss these (“Oh there’s nothing to worry about, vaccination is very safe nowadays”) [27,53]. Instead it is important to fully understand parents’ concerns and motivations using open questions and empathic responses [27]. Although health professionals may be reluctant to encourage questions [27,54], with practice, targeted questions allow health professionals to tailor their discussions [51].

Giving information is an integral part of the immunisation encounter. Here, the skills for efficient information provision are useful - primarily ‘signposting’ and ‘chunking and checking’ (see 1 5, 6 and 7 for examples) [49]. Signposting is the skill of clearly indicating to the parent (or patient) the different phases of the consultation. Chunking and checking refers to the provision of information in small chunks followed by checking the person’s understanding. This technique contrasts with the common practice of providing much larger amounts of information before checking, which can lead to information overload. 

**Table 4:** Parental position, with the recommendations for each group

**Parental position**	**Key indicators**	**Goal**	Strategies*
			**See also Table**3
*Unquestioning acceptor*	Present for vaccination when it is due	Child vaccinated and parent positive about decision	Build rapport
*Cautious acceptor*	Child is fully vaccinated to date		Accept questions and concerns
			Use verbal and numerical descriptions of vaccine and disease risks
			Explain common side effects and rare, important risks
			Aim to keep discussion brief but flexibly addressing parent’s needs
*The hesitant*	Present on time or slightly late	Child vaccinated and parent accepts decision	Use guiding style
*Late or selective vaccinator*	Child is fully or partially vaccinated	This group may need most time but are most likely to change behaviour	Provide risk and benefit information (as above)
	Present late		Use decision aids and other quality information tools
	Child is partially vaccinated		Book another appointment to re-visit discussion
*Refuser*	Present for another reason. Subject of vaccination may have to be raised by health professional.	Parent prepared to think about vaccination and attend clinic for further discussion	Avoid scientific ‘ping pong’ – debating back and forth about vaccination.
	Child is partially or completely unvaccinated	Feels concerns heard and not critical of providers	Ask about importance of protecting child against infectious disease and confidence in the vaccine and respond accordingly
		Parent is aware of the risks of not immunising the child	Explore receptivity to an individualised schedule
			Aim to keep discussion brief but leaving door open to further discussion if parent is moving towards considering vaccination
			Offer attendance at special clinic^†^

* Most strategies are applicable to all groups but located beside those with the most relevance.

† Specialists in some countries offer clinics for children who have experienced an adverse event following immunisation [72].

**Table 5:** Example of dialogue with the unquestioning or cautious acceptor parent

**Health professional:**	Hello Mrs Cheung. I understand you have brought Lily for her vaccinations today.
**Mother:**	Yeah, that’s right.
**Health professional:**	Hello, Lily. OK, have you read the leaflet about the injections? I’d be happy to share with you more information about vaccination. (*build rapport, seek questions and concerns*)
**Mother:**	Well only one thing. She had a slight cold last week, she seems to be over it now but I just wondered if it was safe.
**Health professional:**	She’s back to her normal self now?
**Mother:**	Yes she is
**Health professional:**	Then it is safe for Lily to have them today. (*pausing to allow mother to interject if she has questions and observing body language*) We are vaccinating her against measles, mumps and rubella, Hib, meningococcal C disease and pneumococcal disease* – all serious diseases which have been made much rarer through vaccination programs. It will be three injections and I will give her two in one arm and one in the other arm. They may upset her for a few moments but most children settle straight away after some comforting and 90% don’t have any other side effects at all (*positive framing of risk using percentages*). If there is a problem, the commonest thing is a slightly sore arm that will last for a few days and then settles (*pause to allow questions or clarification* – *chunking and checking*).
**Mother:**	OK – anything else?
**Health professional:**	One of the vaccines contains a small amount of weakened measles, mumps and rubella viruses which stimulate Lily’s immune system to respond and develop protection to these infections. That means she may have some mild symptoms of measles, such as a rash and a fever, and she may feel a bit off-colour 7 to 11 days after the vaccine.*(pause)* About 3 weeks after the vaccine, she may get a mild form of mumps, with swelling under her jaw. But this is less common and happens in only about 1% of children *(qualitative and quantitative risk estimates*). These symptoms are not infectious so she can’t pass them onto to anyone else and they usually go away after 1 to 2 days. The side-effects of the vaccine are usually mild and they are milder than the risks of having measles, mumps or rubella diseases. If you have worries afterwards, bring her back to the clinic and we can check her over. How does that sound? (*structured information using chunks and checks and unbiased expectation of consent*)
**Mother:**	Fine, yeah, that’s OK.

**Table 6:** Example of dialogue with the hesitant parent

**Health professional:**	Good morning Mrs Wilkinson. I understand you have brought Robbie for his first infant vaccinations today.
**Mother:**	That’s right.
**Health professional:**	OK, have you read the leaflet about the injections? What questions are on your mind? (*build rapport, seek questions and concerns*)
**Mother:**	Well, I’m pretty nervous – he seems so young.
**Health professional:**	You sound quite worried (*empathic response*), let’s talk it through together, tell me what you are concerned about? (*further building rapport and eliciting concerns*)
**Mother:**	One of the mums in my mothers’ group said that one of the injections has got five ingredients and that’s too many for their immune systems to cope with. He does seem so young to be having injections against all these diseases at once. Won’t it make him ill?
**Health professional:**	OK, we can talk about this (*guiding*) but do you have other worries as well? *(eliciting further concerns)*
**Mother:**	Well I read also that they can get a sore leg afterwards, so that’s another worry.
**Health professional:**	(*pausing to allow mother to interject if she has questions and to observe body language*) Right, let’s talk about the five ingredients and then we can talk about the chances of getting a sore leg (*signposting and structuring of explanation*). You’re right that the injection has got five ingredients which would protect Robbie from the diseases called diphtheria, tetanus, whooping cough, polio and *Haemophilus influenzae* b (Hib)*.* It seems a lot doesn’t it *(empathic response).* Children, even newborn babies, have to deal with enormous amounts of bacteria and other foreign material every day, and the immune system responds to each of these in various ways to protect the body. Babies’ immune systems can handle this, and the vaccines these days are so refined that babies can easily cope with several vaccines in one go. (*chunk of information provided followed by pause for mother to raise further questions and health professional to observe mother’s body language*).
**Mother:**	OK, and will he get a sore leg?
**Health professional:**	Most children don’t have any reaction at all, other than having a cry with the injection, and even then they generally settle really quickly with a cuddle and some comforting words from mum (*empowering*). It’s true that a small number of children, about 10%, or 1 in 10, can get a redness or a sore area where the needle goes in *(acknowledging)* – but these reactions don’t usually distress the child, and only last a couple of days, then go away. So what I ask mothers to do is to watch their child and if they are concerned bring them back to the clinic so we can check them over. How does that sound? (*avoid being overly persuasive, positive framing of risk*)
**Mother:**	Is there anything in particular I should watch for?
**Health professional:**	Robbie may be a bit unsettled for a day or so after his injection but he shouldn’t be ill with it. The leaflet tells you about what to look out for and what to do if you are concerned.
**Mother:**	Thanks – I’m still a bit nervous but I think we should get it done.

#### What to include in discussions with parents

It is important to communicate risk effectively [56]. It is recommended that health professionals give information about common but minor side effects, and rare but serious ones [57]. Written materials, web links, or decision aids given prior to, or used during, the consultation can be helpful [58-60]. In a recent UK survey 156 primary health care professionals viewed the inclusion of a web link for an online MMR decision aid contained in a parents’ MMR pre-vaccination invitation letter as an appropriate way to support parents coming to the consultation [61]. Written resources may be available in electronic/online or paper format. These vary widely between countries and clinicians should be familiar with how to locate them.

Risk communication is best tailored to individuals. In general terms outcomes are better understood when they are specified and when their probability is given in numbers (e.g. 1 in 1000) although some may prefer words [62]. When presenting probabilities, there remains conflicting evidence over whether natural frequencies (e.g., 1 in 100) or percentages are preferable [63]. A recent study concluded that percentages may be better understood than natural frequencies [64]. To avoid confusion, a consistent denominator should be used when presenting event rates for comparison [65]. Visual representations of probability have also been recommended and are commonly used in decision aids [59,61].

Specific information is most helpful when it is timely, consistent, relevant, up to date, and, where available, local. Parents should also be advised about how to manage the common side effects of vaccinations and how to seek help if they have further concerns [66].

#### A tailored approach

Evidence from other areas of healthcare practice suggests adapting the principles of motivational interviewing. This is a form of communication that uses a guiding style, rather than a directing style, for discussions where there is ambivalence and resistance to change [51]. Motivational interviewing involves asking questions that clarify an individual’s responsiveness to change and elicits their own motivations for change. The method has demonstrated effectiveness in a range of health behaviours [67]. In this particular context, the ambivalence and resistance to change relates to whether or not a parent should have their child vaccinated rather than focusing on a behaviour such as quitting smoking.

It should also be borne in mind that motivational interviewing builds upon the Transtheoretical Model [68]. This is a framework for understanding the process of behaviour change where individuals may pass through five stages: *precontemplation*, where they are not considering change; *contemplation*, where they seriously consider change; *preparation*, where they plan and commit to change; *action*, where they make a specific behavioural change which if successful, leads to *maintenance* of that behaviour, the fifth stage.

Accordingly, Table 4 strategies tailored to the parent’s stage. The majority of parents are in the *action* and *maintenance* stage (cautious or unquestioning acceptors). Some will be *contemplating* or even *preparing* to immunise – what we describe as ‘hesitant’ parents. Late or selective vaccinators, who are willing to have some vaccines, may also *contemplate* full vaccination if guided by a health professional [5]. ‘Vaccine refusers’ are usually in the *pre-contemplation* stage where they are not considering vaccinating at all. It is unrealistic to expect such parents to move to the *action* stage at one visit. However, the goal may be to guide them toward *contemplating* vaccination. This would be done by asking permission to discuss; encouraging them to explore the pros and cons of their decision; and eliciting their own possible motivations to change (Table 4).

Tables 5, 6 and 7 give examples of suggested dialogue for unquestioning and cautious acceptors, hesitant, and refusing parents. For late or selective vaccinators, strategies can apply from those suggested for hesitant and refusing parents. A parent’s starting position can be clarified with initial questions (How do you feel about the vaccinations?) and observation of their body language.

It may also be possible to flag specific questions or concerns for discussion prior to the consultation. For example, a question prompt sheet for parents to use in consultations about MMR vaccination was positively evaluated by 46 parents in a UK study [69]. Specifically parents reported that the prompt sheet enabled them to feel confident in ‘raising the issue of MMR’ with their GP or nurse.

The goals for the consultation will vary according to the parent’s position. Health professionals should avoid a mismatch between the parent’s expectations and needs and their own assumptions. For example, a ‘hesitant’ father may be planning only to obtain information but feels he is being pressured to vaccinate. The cost may be loss of his trust and a subsequent unwillingness to return. Similarly, a ‘refusing’ mother might be approached by a health professional who is intent on changing her mind [70]. This ‘righting reflex’ is the natural response of health professionals to instinctively ‘put right’ healthcare problems rather than finding out patients’ concerns or points of view. It may lead to an adversarial position and further entrench the parent’s views, closing the door to any future possible gains [71]. In this situation, a better goal would be to build a rapport that may lead to willingness for further discussion or partial vaccination. Vaccine-refusing parents may be willing to consider an alternative schedule and may be willing to hear how to recognise and respond early to signs that their child may have a vaccine-preventable disease (Table 4). 

**Table 7:** Example of dialogue with the vaccine-refusing parent

	*There is a discussion about Oliver’s upper respiratory tract infection then:*
**Health professional:**	Do you mind if we take a moment to talk about Oliver’s vaccinations?
**Mother:**	Ah, yes, we did some research into it and decided not to vaccinate him.
**Health professional:**	OK, can I just talk it through so I understand your decision? (*asking permission to discuss and use of a guiding style*)
**Mother:**	Yeah, OK.
**Health professional:**	To start with can I just ask you how important you think it is to get Oliver protected from the diseases vaccines are designed to prevent? (*assessing importance*)
**Mother:**	Well, mostly the diseases aren’t that much of a problem in healthy children and we keep Oliver very healthy with a good diet, organic food, and plenty of fresh air.
**Health professional:**	You’re right, most children will overcome illnesses without too much of a problem (*acknowledging*)*.* Unfortunately, there are still children that get pretty sick with these diseases, and sadly a significant number of children end up in hospital with complications from the disease. With measles, for example, 9 in every 100 children get pneumonia and some need to go to hospital (*pause*).
**Mother:**	I didn’t know that.
**Health professional:**	Yes, it can still be a serious problem. Could I ask now how confident you are that the vaccinations are safe? (*assessing confidence)*
**Mother:**	I’m not all confident in them being safe.
**Health professional:**	What have you heard? *(exploring)*
**Mother:**	Well on one internet site it said that children can get brain damage and all kinds of problems after vaccination. And the drug companies try to cover it up.
**Health professional:**	That sounds frightening (*empathic response*). Which vaccines are you most concerned about? (*eliciting specific concerns*)
**Mother:**	The MMR one because it can cause autism.
**Health professional:**	I understand you are concerned about vaccinations *(building rapport by accepting rather than rebutting concerns)* but I’d just like to give you my view if that’s OK? *(Mother nods.)* Although there has been some research that raises concerns about vaccine safety, each time a concern comes up, new research is done to check whether the results are consistent or not. The vaccines that we use are very safe and serious side effects are very rare. Would you like to look at the MMR vaccine decision aid which can help you weigh up the risks of the vaccine and the diseases? (*respecting autonomy, offering information*)
**Mother:**	Well, I guess I could have a look but I’m still pretty cautious about Oliver getting these jabs.
**Health professional:**	Well, take a look at the decision aid and then if you like, come back to the clinic for another talk. We have a clinic each Tuesday and I’ll be here most weeks. Would you like to come back in two weeks? (*leaving door open to further discussion*)
**Mother:**	OK thanks.

## Discussion

As vaccine preventable diseases become less common, parents in industrialised countries appear to be expressing more concerns about the safety and necessity of vaccines. This could lead to a decline in vaccination uptake rates to a level which allows the diseases to re-emerge and become significant health problems [73].

Health professionals have to juggle the need to consider the population at risk of the disease (particularly if vaccination rates drop) alongside addressing the needs of the particular parent who is raising concerns about what to do for their particular child. These concerns centre on an increasing number of vaccines given to children, their safety, composition and necessity.

Since interactions with health professionals provide a focal point for parents’ concerns to be expressed, it is important that communication during these interactions is effective [26]. The parental positions described in this paper act as a starting point for health professionals to choose the most appropriate communication strategy. Naturally these will vary according to the parent’s individual needs and circumstances.

The literature review informing the parental positions found only three papers providing a spectrum of attitudes to vaccination. While there is a vast literature exploring attitudes to vaccination among parents [3,7,74,75], we sought to identify only studies that would provide a spectrum of attitudinal positions that would theoretically account for all parents.

However, the spectrum described does simplify what is often a complex process of decision making which may involve parents moving between positions over time. Indeed, as noted in the wider literature, parents’ decisions are also made in a broader context of beliefs about a child’s health, personal experiences, perceived norms, and trust in health systems and professionals [12,29,76]. While the parental positions were developed from three US studies, we have applied our knowledge and experience from other countries to their modification.

The approximate proportions of each parental group are estimates from population-based surveys and will vary over time, within regions, and between practices. For example, the estimate for the percentage of parents completely refusing all vaccines is given as less than 2% based on population data but there are clusters of much higher refuser rates in specific localities [43,44,77]. Nevertheless, giving ranges for the sizes of the groups may assist health professionals and programme coordinators in planning for targeted information and strategies.

We have proposed an approach to communication that encourages questions and employs a guiding rather than directing style. The reality of busy clinical environments can act as a disincentive for health professionals to actively seek out questions and concerns [54]. However, the framework we propose may ensure that consultations are more time efficient because it provides a structure to more rapidly identify the parent’s position on vaccination, the most appropriate goals for that consultation, and the parent’s specific information needs. Practised interviewing techniques enable the health professional to quickly focus the discussion on the specific concerns of the parent. In interactions with vaccine-refusing parents, some health professionals attempt to change the parent’s mind [70]. As this goal is usually unrealistic, the consultation can become long and difficult and result in an impasse. Having more realistic goals will facilitate a more satisfying and time efficient discussion which may then be followed-up as needed.

Communication strategies to date have lacked clear evidence of efficacy in vaccination settings. We have described a framework for talking with parents about vaccination. It is informed by evidence and acceptable to the health professionals involved in the formative evaluation sessions, but now needs to be more fully evaluated. This may involve group or individual training of health professionals who undergo assessment using standardised patients and validated scales that measure quality of communication [78]. To measure effectiveness of the framework against the aims described above (satisfaction, self-efficacy, decision quality and vaccination uptake), a randomised controlled trial delivered at cluster (e.g., GP practice) or individual level would then establish its effectiveness compared with ‘usual care’.

## Conclusion

Good communication is part of a suite of measures needed to maintain high uptake of child vaccines. Strategies must also continue to address barriers such as access to healthcare and provider factors [79-81]. Nevertheless, there is an urgent need to build an evidence base which informs vaccine communication, given that the parent–provider interaction remains integral to maintaining public confidence in vaccination.

## Competing interests

All authors have completed the Unified Competing Interest form at http://www.icmje.org/coi_disclosure.pdf and declare: no support from any organisation for the submitted work; no financial relationships with any organisations that might have an interest in the submitted work in the previous three years, no other relationships or activities that could appear to have influenced the submitted work.

## Authors’ contributions

JL and PK had the idea for the article, CJ and FC performed the literature search, JL, PK, CJ, FC, HB and GR developed the framework and wrote the article. JL is the guarantor. All authors read and approved the final manuscript.

## Pre-publication history

The pre-publication history for this paper can be accessed here:

http://www.biomedcentral.com/1471-2431/12/154/prepub
